# The Density of CD10 Corresponds to Commitment and Progression in the Human B Lymphoid Lineage

**DOI:** 10.1371/journal.pone.0012954

**Published:** 2010-09-23

**Authors:** Michiko Ichii, Kenji Oritani, Takafumi Yokota, Qingzhao Zhang, Karla P. Garrett, Yuzuru Kanakura, Paul W. Kincade

**Affiliations:** 1 Immunobiology and Cancer Program, Oklahoma Medical Research Foundation, Oklahoma City, Oklahoma, United States of America; 2 Department of Hematology and Oncology, Osaka University Graduate School of Medicine, Osaka, Japan; New York University, United States of America

## Abstract

**Background:**

Requirements for human B lymphopoiesis are still poorly understood, and that has hampered investigation of differentiation events. For example, there are few cell surface antigens that can be used as milestones of lineage progression. The CD10 ectoenzyme is one such marker and has been used to define CLP, but we found substantial tissue specific variations in CD10 levels, and there was no information about how that corresponded to differentiation options.

**Methodology/Principal Findings:**

The aim of the present study was to use recently developed culture methods to assess the nature and differentiation potential of progenitors sorted according to CD10 density from umbilical cord blood (CB), adult bone marrow (BM) or G-CSF mobilized peripheral blood (PB). Many CD34^+^ cells in BM express high levels of CD10, while low or low/negative CD10 densities were found on CD34^+^ cells in CB or G-CSF mobilized PB, respectively. The relative abundance of CD10^Lo^ versus CD10^Hi^ cells only accounts for some CB versus BM differences. Almost all of the CD34^+^ CD10^Hi^ cells expressed CD19 and lymphocyte transcription factors and corresponded to loss of myeloid potential. A high degree of immunoglobulin D_H_-J_H_ gene rearrangements was characteristic only of the CD10^Hi^ subset. In contrast, the CD34^+^ CD10^Lo^ progenitors efficiently produced plasmacytoid and conventional dendritic cells as well as myeloid cells. These findings suggest a positive correlation between CD10 density and degree of differentiation. Although freshly isolated CD34^+^ CD10^Hi^ cells were in cycle, those from CB or BM expanded poorly in culture, suggesting regulators of populations remain to be discovered.

**Conclusions/Significance:**

Steps in human B lymphopoiesis have not been sufficiently studied, and we now show that increased CD10 expression corresponds to differentiation potential and stage. CD34^+^ CD10^Hi^ progenitors are obviously in the B lineage but may have progressed beyond the point where they can be expanded in culture.

## Introduction

Common lymphoid progenitors (CLP) were originally defined in mice as lineage marker negative, IL-7Rα^+^, Sca-1^+^, c-Kit^Lo^ cells that appeared to be largely restricted to the production of B, T and NK cells [1]. The original CLP subset has been subdivided and re-defined in multiple ways, and the potential of these cells to produce small numbers of non-lymphoid cells is still debated [2]–[7]. Moreover, a picture is emerging of asynchronous expression of lymphoid genes and markers, with gradual restriction of differentiation options [8], [9].

Although the CD10 ectoenzyme is not expressed on murine progenitors, it has been used in the definition of human CLP [10]–[12]. The current consensus is that CD10 is expressed on early, pro-, and pre- B cells while levels are down-regulated after development to mature B cells. Other markers exploited in studies of human but not murine CLP include CD45RA, CD7 and CD38 [13]–[15]. In addition, multiple differentiation pathways can result in production of similar cells such as T and B lymphocytes [16]–[18]. There are additional reasons comparisons between murine and human lymphoid progenitors are difficult. For example, IL-7 is not essential for human B lymphopoiesis, and no factors have been discovered that support efficient B lymphoid lineage progression in culture [19]. For these and other reasons, results obtained in studies conducted with bone marrow from young adult mice are not readily comparable to those using human umbilical cord blood (CB), adult bone marrow (BM) or G-CSF mobilized peripheral blood (G-PB).

Allogeneic hematopoietic stem cell transplantation is a curative treatment for patients with hematopoietic malignancies and marrow failure syndrome [20]–[22]. However, the composition of the donor inoculum has a substantial influence on transplantation related mortality, occurrence of graft-versus-host disease and relapse of diseases. For example, high numbers of infused CD34^+^ CD19^+^ B progenitors were associated with lower incidences of acute GVHD and transplant related mortality [23]. More information about stages of B lymphopoiesis and how they vary in different stem cell sources could improve clinical outcomes.

We have now found that human CD34^+^ cells from CB, BM and G-PB express a wide range of CD10 densities. Increasing levels of CD10 corresponded to expression of lymphoid related transcription factors and markers, as well as loss of proliferative potential in two recently developed culture systems. Moreover, myeloid differentiation potential was not completely lost until CD10 levels were very high. Complete restriction to a B lineage lymphoid fate may be a very late event in human lymphopoiesis.

## Results and Discussion

### Hematopoietic progenitors in different sites vary with respect to CD10 density and composition

**Figure 1:**
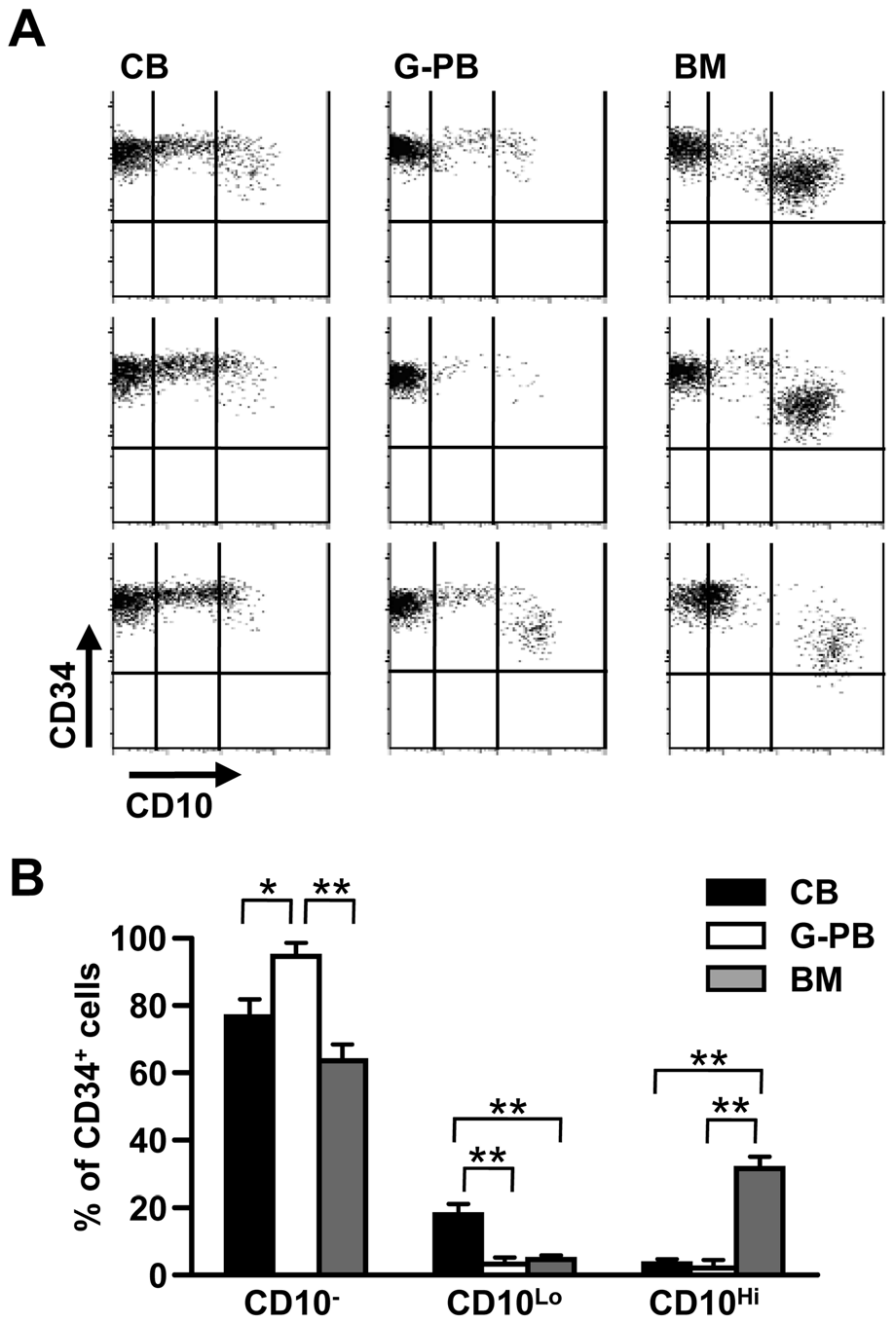
Densities of CD10 vary among human CD34^+^ cells isolated in different ways. CD34^+^ cells enriched from CB, G-CSF mobilized peripheral blood (G-PB) and BM were stained with PE-CD10 and APC-CD34 for flow cytometry analysis. (A) They were partitioned into three categories according to CD10 levels. (B) Incidences of CD34^+^ CD10^−^, CD34^+^ CD10^Lo^, and CD34^+^ CD10^Hi^ progenitors in these preparations are shown. Similar results were obtained in three independent experiments utilizing specimens from four different donors. Statistical significances were determined by unpaired two-tailed *t* test analysis: *, *p*<0.05 and **, *p*<0.01.

We developed a new co-culture system where CD34^+^ hematopoietic cells are sorted and placed on monolayers of human mesenchymal stem cells along with stem cell factor (SCF) and Flt3 ligand (see Materials & Methods). Under these conditions, CD10^+^ CD19^+^ lymphocytes emerged within 2–3 weeks. As noted in previous studies, the source of the progenitors was an important variable, and the efficiency was reproducibly higher in cultures initiated with CB (Figure S1) [13], [24], [25]. Differences in the composition of CD34^+^ cell suspensions might account for this, because it has been found that CD10^+^ progenitors from CB and BM are functionally equivalent [14], [26]. That is, we considered that CB could be enriched with respect to CD10^+^ CLP. To our surprise, BM contained more CD10^+^ cells, and the average density of this marker was much higher (Figure 1).

**Figure 2:**
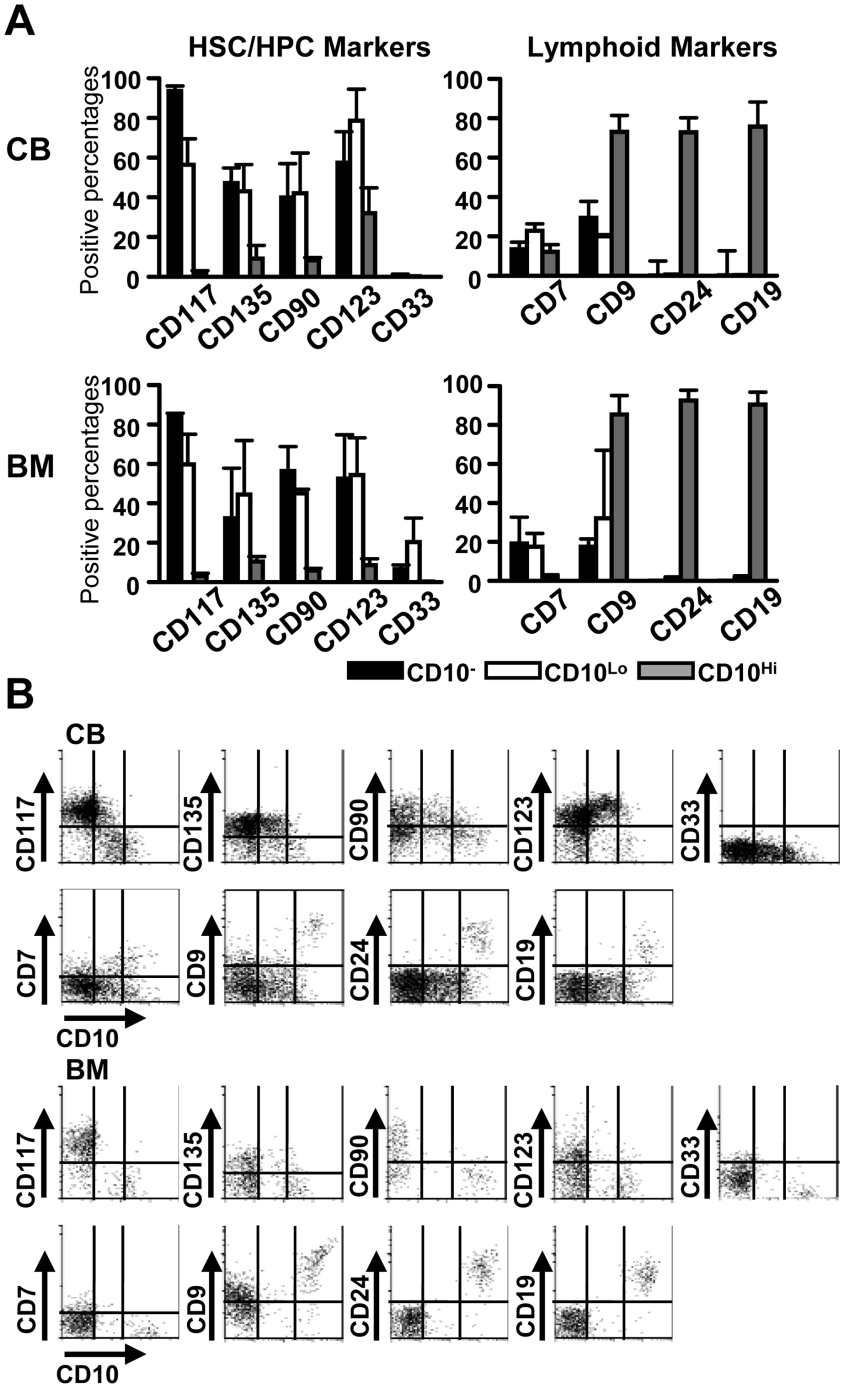
High levels of CD10 correspond to progression in the B lineage. (A, B) CD34^+^ cells from CB and BM were stained with CD10 and CD34 as well as the indicated antibodies, and analyzed by flow cytometry. The CD34^+^ cells were gated according to CD10 levels and percentages of cells positive for each marker are shown. Similar results were obtained with cells from at least three different donors in three separate experiments.

CD34^+^ cells were then enriched in samples representing three sources and flow cytometry was used to discern expression of other markers associated with early events in human hematopoiesis (Figure 2). Down-regulation of CD117/c-Kit occurs with progression from the stem/multipotential progenitor stage, and this receptor for SCF was entirely lacking in the CD10^Hi^ subset of CD34^+^ cells. Similarly, the CD135 receptor for Flt3 ligand, the CD90/Thy-1 antigen and the CD123 receptor for interleukin 3 were low on CD10^Hi^ cells. Only small numbers of CD10^Lo^ cells in BM displayed the CD33 myeloid marker. The deficiency of stem and early progenitor markers was offset by expression of CD9, CD24 and CD19 lymphoid associated antigens on CD10^Hi^ CD34^+^ cells. Although the incidence of CD10^+^ cells in G-PB was low, staining patterns with the other markers were very similar to CB and BM (data not shown). In fact, when CD34^+^ cells were resolved according to CD10 density, patterns of all other markers were remarkably similar, regardless of tissue of origin. As is the case with murine studies, there is no standardized definition of human CLP, and a series of markers have been used to resolve lymphopoietic progenitors. CD34^+^ progenitors with low CD10 most closely resemble the original CLP described by Galy and colleagues [11]. CD24 was absent from CD34^+^ CD10^Lo^ progenitors suggesting they are not dedicated to the B lineage [16].

**Figure 3:**
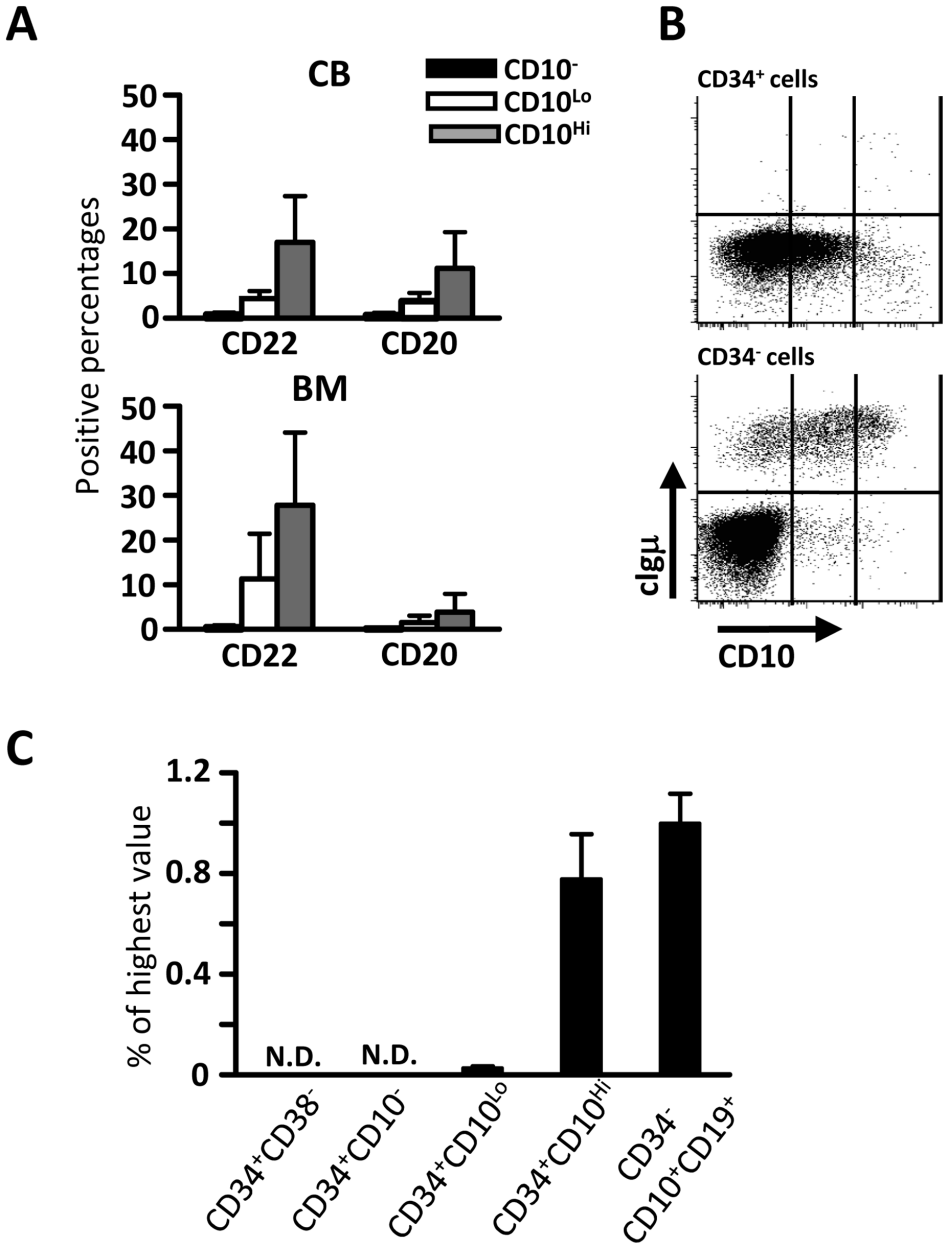
D_H_-J_H_ rearrangements\cur in progenitors with high CD10 densities. (A) Expression patterns of CD22 and CD20 on CD34^+^ CD10^−^, CD34^+^ CD10^Lo^, and CD34^+^ CD10^Hi^ progenitors derived from CB and BM were analyzed using flow cytometry. (B) After staining CD34^+^ or CD34^−^cells from CB and BM with CD10 and CD34 antibodies and fixing, they were stained with cytoplasmic Igµ. The data is representative of results obtained with cells from at least three diffferent donors in three separate experiments. (C) The indicated fractions were sorted from CB, and genomic DNA was extracted from 10^4^ cells. Sorted CD34^+^ CD38^−^ and CD34^−^ CD10^+^ CD19^+^ cells were used as negative and positive controls, respectively. C/Ebpα specific PCR reactions were used to determine input quantities. The results are normalized as percentages of peak values for each of the genes and are representative of that obtained in three independent experiments, using either CB or BM. N.D.; not detected.

To obtain more detailed information about B lineage progression, we studied CD22, CD20 and cytoplasmic Igµ (cIgµ) expression with each of the three subsets (Figure 3A, B). Approximately, 20% of CD34^+^ CD10^Hi^ cells expressed CD22, and 5–10% of them were CD20^+^. There were very few cIgµ positive cells among CB and BM CD34^+^ fraction (less than 2% of CD34^+^ cells). Zelm et al. reported that incomplete D_H_-J_H_ rearrangements were initiated in CD34^+^ CD22^+^CD19^−^ cells, and V_H_-DJ_H_ rearrangements mainly occurred in CD34^−^ CD10^+^ CD19^+^ CD20^−^ cells [27]. *IgK/IgL* gene rearrangements were initiated after CD34 down-regulation and expression of cIgµ (in CD34^−^ CD10^+^ CD19^+^ CD20^−^ cells). Therefore, we analyzed immunoglobulin D_H_-J_H_ gene rearrangements by quantitative PCR in subsets separated on the basis of CD10. The human IgH locus contains 66 rearrangeable V_H_, 27 D_H_, and 6 J_H_ genes, so we designed probes to detect the predominant D_H_3–10 gene segment and the J_H_4 gene segment. This particular D_H_-J_H_ rearrangement most frequently occurs in humans [28]. Contrary to our expectations, Ig gene rearrangement was a late event, with high levels found only in the CD34^+^ CD10^Hi^ subset. Similar results were obtained in three independent experiments using cord blood or adult marrow derived progenitors.

Hematopoietic cells in humans and mice differ with respect to many cell surface markers [1]–[7], [11]–[16], [27], [29], complicating extrapolation between the two (Figure S2). For example, freshly isolated murine lymphoid progenitors do not express CD10 [10]. In contrast, we now show that levels parallel lineage progression in humans. Our results suggest that CD10 levels represent an important variable, increasing coincident with initiation of Ig gene rearrangement and before uniform expression of either CD20 or CD22. Subsequent experiments were aimed at determining if it corresponded to differentiation and lineage restriction.

### CD10 density increases with expression of lymphoid lineage transcription factors, but loss of myeloid potential by CD10 bearing progenitors is a late event

**Figure 4:**
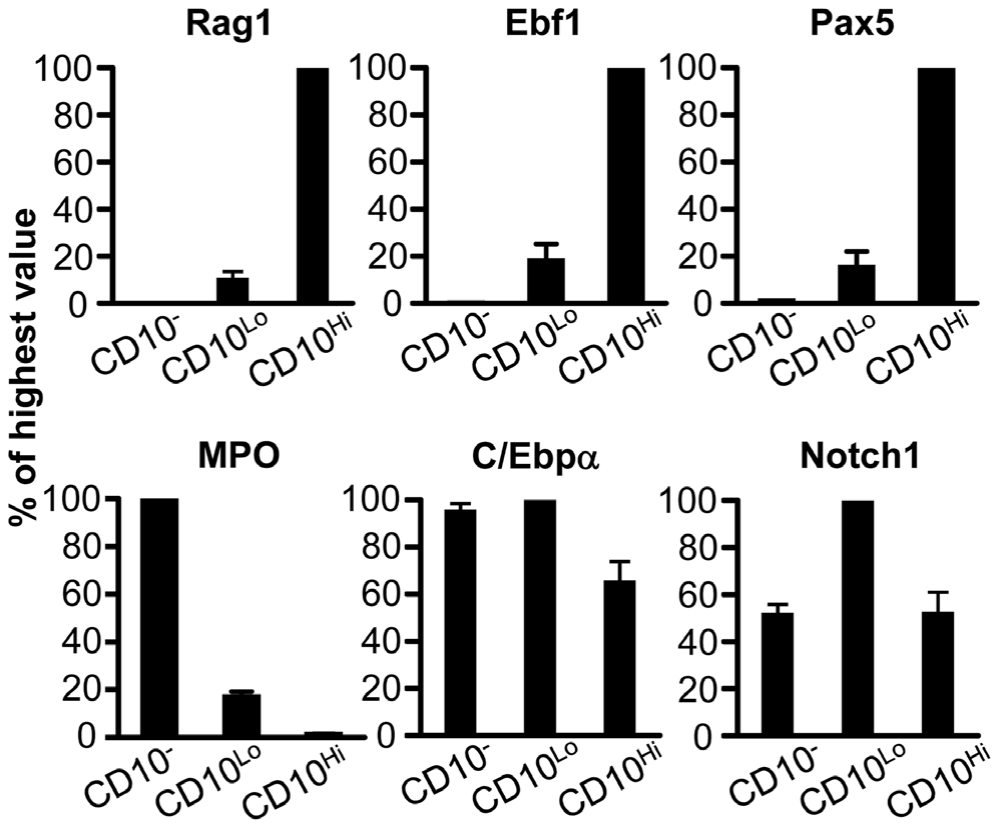
Gene expression patterns suggest that lymphoid commitment parallels acquisition of CD10. CD34^+^ CD10^−^, CD34^+^ CD10^Lo^ and CD34^+^ CD10^Hi^ cells were sorted from BM. mRNA was extracted from each subset, and quantitative RT-PCR was conducted. The results are normalized as percentages of peak values for each of the genes and are representative of three independent experiments. Similar results were obtained in three experiments, using either CB or BM.

Consistent with changes in proteins detected by flow cytometry, transcripts for the RAG-1recombinase enzyme markedly increased with CD10 up-regulation (Figure 4). The Ebf1 and Pax5 transcription factors are essential for B lymphopoiesis, and the corresponding transcripts increased in parallel with CD10. In contrast, expression of the myeloid associated myeloperoxidase (MPO) gene was down-regulated with CD10 acquisition. All of these three subsets expressed C/Ebpα and Notch1. Results were very similar when CB rather than BM was used as a source of progenitors (data not shown). Hystad and colleagues performed microarray analyses on human B lineage progenitors separated according to presence or absence of markers [29]. Comparing their developmental milestones to our RT-PCR results indicates that CD34^+^ CD10^Lo^ cells are relatively primitive, but primed for lymphopoiesis. Abrupt increases in CD10 transcripts were found in another study to correspond with Ig gene rearrangement activity [27].

**Figure 5:**
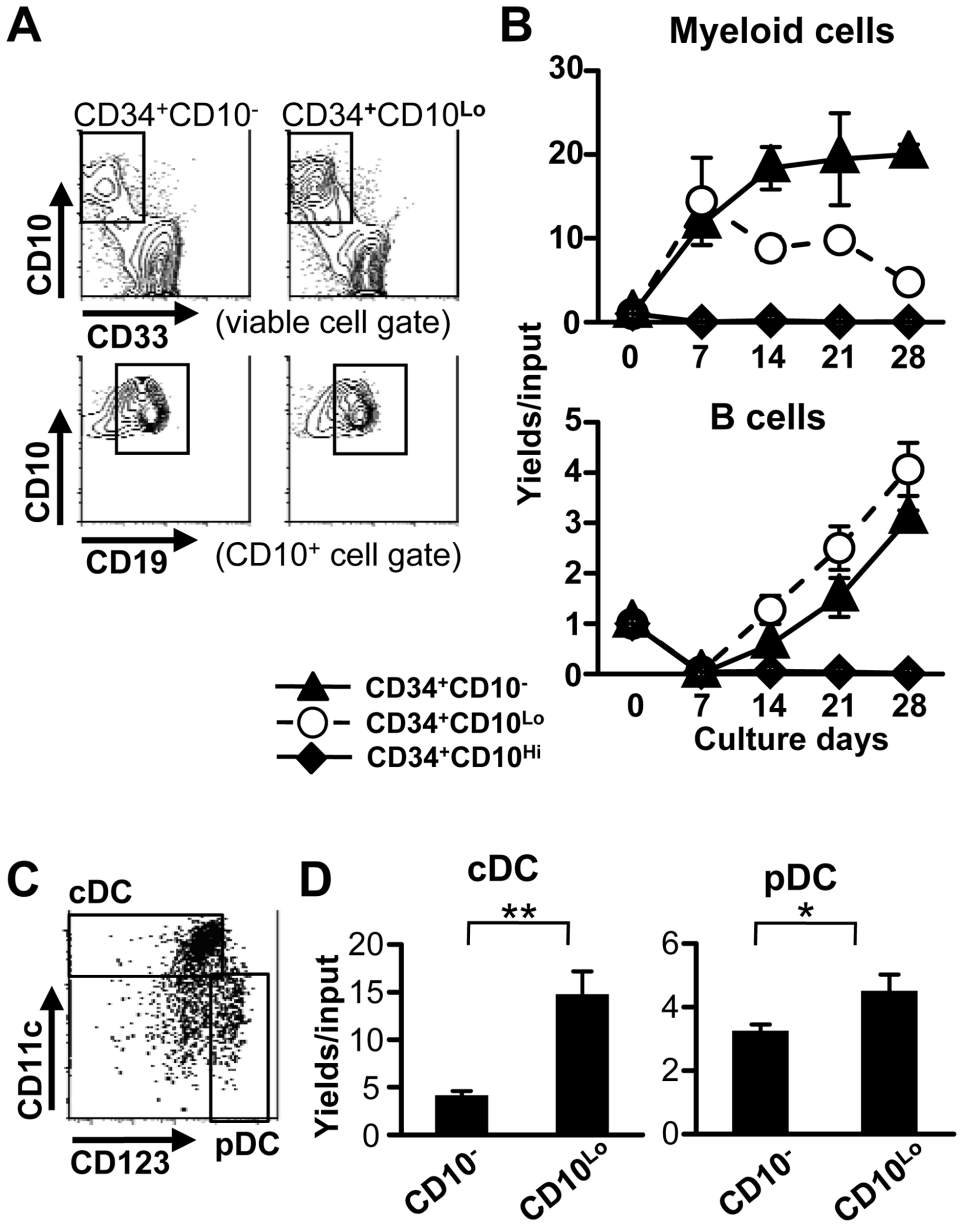
Both CD34^+^ CD10^−^ and CD34^+^ CD10^Lo^ subsets can generate DC and myeloid lineage cells. (A, B) CD34^+^ CD10^−^, CD34^+^ CD10^Lo^ and CD34^+^ CD10^Hi^ cells were sorted from CB and cultured with hMSC in the presence of SCF and FL for 4 weeks. Representative flow cytometry results are shown for a 4 week co-culture experiment performed with pooled cord blood specimens (A). The cultured cells were collected weekly, and numbers of generated CD33^+^ myeloid cells (B, upper panel) and CD10^+^ CD19^+^ B cells (B, lower panel) were determined. Similar results were obtained in three independent experiments. (C, D) CD34^+^ CD10^−^ and CD34^+^ CD10^Lo^ CB cells were cultured with hMSC in the presence of SCF and FL for 2 weeks. Cells recovered and gated as CD14^−^, CD34^−^ and CD19^−^ were characterized by flow cytometry (C), and numbers used to calculate yields per input cells (D). Similar results were obtained in three independent experiments. Statistical significances were determined by unpaired two-tailed *t* test analysis: *, *p*<0.05 and **, *p*<0.01.

Interestingly, the experiments described above revealed that CD34^+^ progenitors with CD10 still had C/Ebpα gene expression, and CD34^+^ CD10^Lo^ progenitors expressed MPO at a low level, as reported before [27], [29]. This raises important questions concerning their differentiation potential. Therefore, we sorted CD34^+^ cells from CB and carefully assessed their progeny over four weeks of culture (Figure 5A, B). A substantial wave of CD33^+^ myeloid cells was observed in cultures initiated with CD34^+^ CD10^Lo^ progenitors, and sustained myelopoiesis resulted in remaining cultures of CD10^−^ stem/progenitors. Human mesenchymal stromal cells were used for these experiments because of their ability to simultaneously support B, dendritic and myeloid differentiation. Therefore, we recovered CD14^−^ CD19^−^ CD11c^+^ CD123 ^Lo/-^ conventional dendritic cells, as well as CD14^−^ CD19^−^ CD11c^−^ CD123^Hi^ plasmacytoid dendritic cells from 2 week co-cultures (Figure 5C, D). We conclude that CD10^Lo^ progenitors are not lymphoid lineage restricted.

### CD34^+^ CD10^Hi^ progenitors are strictly lineage-committed, but have diminished ability to expand in culture

**Figure 6:**
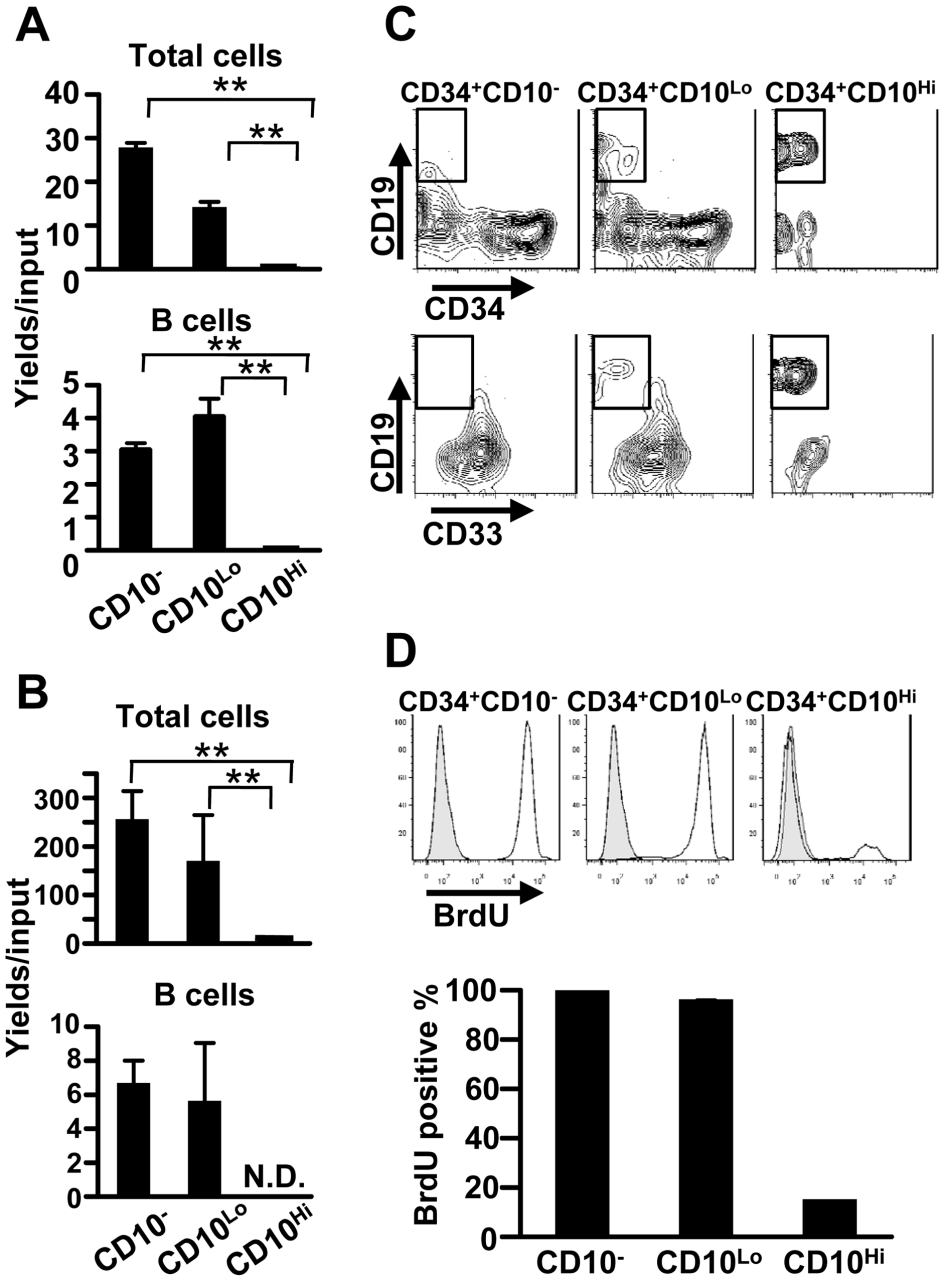
CD34^+^ CD10^Hi^ progenitors can differentiate but not proliferate in vitro. (A) CD34^+^ CD10^−^, CD34^+^ CD10^Lo^ and CD34^+^ CD10^Hi^ cells were sorted from CB and stromal cell co-cultures were maintained with SCF and FL for 4 weeks before flow cytometry analyses. (B) The three categories of CD34^+^ CB progenitors were cultured under stromal cell-free conditions, but with SCF, FL and IL-7 for 3 weeks. Numbers of total and CD10^+^ CD19^+^ cells per input progenitor were calculated. (C) CD34^+^ CD10^Lo^ and CD34^+^ CD10^Hi^ cells were sorted from BM and cultured under stromal cell-free conditions for 1 week. The generated cells were stained with CD19, CD33 and CD34 and were analyzed by flow cytometry. One representative determination is shown. (D) CD34^+^ CD10^−^, CD34^+^ CD10^Lo^ and CD34^+^ CD10^Hi^ cells were sorted from CB and cultured in the presence of SCF, FL, and G-CSF for 4 days. They were pulsed with 10 mM BrdU for the final 48 hours. Similar results were obtained in three independent experiments. Statistical significances were determined by unpaired two-tailed *t* test analysis: **, *p*<0.01. N.D.; not detected.

Given that CD10^Hi^ progenitors express characteristics associated with B lineage lymphocytes, we assumed that they would be potent progenitors when placed in culture. However, yields of total cells and CD19^+^ B lineage lymphocytes were very low at all time points in cultures initiated with CD34^+^ CD10^Hi^ progenitors (Figure 5, 6A). This is despite the fact that these culture conditions are optimized for human B lymphopoiesis [30] and gave good yields with CD10^−^ and CD10^Lo^ subsets (Figure 5, 6A). Late stages of murine B lymphopoiesis are most efficiently supported by stromal cell-free conditions [31], and we recently adapted that approach for human progenitors [32]. However, attempts to propagate them in four-week stromal cell-free cultures were unsuccessful (Figure 6B).

In one-week stromal cell-free cultures, CD34^+^ CD10^Hi^ progenitors gave rise to CD34^−^ CD33^−^ CD19^+^ lymphocytes, but only with poor survival and/or minimal expansion (Figure 6C). In contrast to CD34^+^ CD10^−^ and CD34^+^ CD10^Lo^ progenitors, the CD10^Hi^ subset proliferated poorly during four days of culture and incorporated little BrdU (Figure 6D). This contrasts with a report that some CD34^+^CD19^+^ cells could generate myeloid and erythroid lineage cells [33]. Although CD10^Hi^ progenitors are rare in CB, we were able to determine that they also expanded poorly in these cultures.

**Figure 7:**
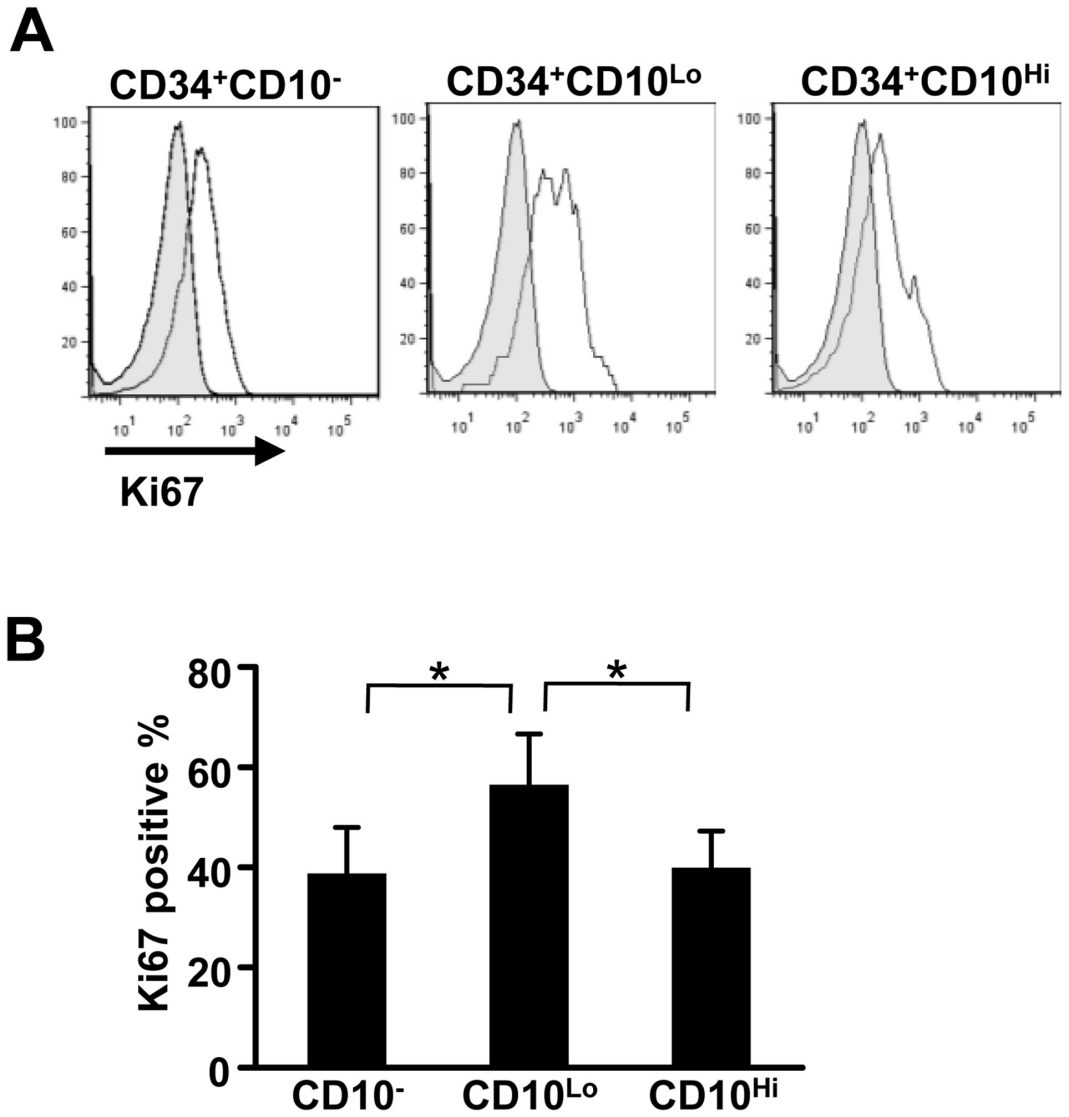
All three subsets include dividing cells when freshly isolated from BM. CD34^+^ CD10^−^, CD34^+^ CD10^Lo^ and CD34^+^ CD10^Hi^ cells were sorted from fresh BM, and Ki67 expression was analyzed on the three subsets. A representative analysis is shown (A) and the data represent mean values with standard deviations from 6 samples (B). Statistical significances were determined by unpaired two-tailed *t* test analysis: *, *p*<0.05.

It is possible that CD34^+^ CD10^Hi^ progenitors are post-mitotic *in vivo*, but staining of freshly isolated BM with the Ki67 proliferation marker revealed that all three subsets include dividing cells (Figure 7A, B). The restricted and rapid generation of CD34^−^ CD19^+^ lymphocytes by CD34^+^ CD10^Hi^ progenitors suggests they represent a lineage committed stage. They could have recently exited cell cycle in vivo. Alternatively, conditions required for their expansion in culture remain undefined.

Definition of factors that regulate human B lymphopoiesis remains an elusive goal, and no cytokine that supports robust human B lymphopoiesis has been found. B lymphopoiesis is not impaired in SCID patients with mutations in the common γ chain required for IL-7 responses, and there is no clear requirement for this cytokine in cultures of freshly isolated progenitors [19], [30], [32], [34]–[36]. On the other hand, there is some evidence that stromal cells and pre-cultured hematopoietic cells can recognize IL-7 [37]–[40]. In addition, a very transient response of human fetal B progenitor cells was reported [19], [41], [42].

To conclude, the composition of umbilical cord blood, G-CSF mobilized peripheral blood and adult bone marrow is markedly different with respect to lymphopoietic cell subsets. Our results show that CD10 density increases in parallel with expression of B lymphoid lineage genes and surface markers on CD34^+^ progenitors. This parameter also corresponds to loss of potential to generate myeloid and dendritic cells in culture. Complete restriction to a B lineage fate appears to be a late event.

## Materials and Methods

### Origin and isolation of cells

CB cells were collected from healthy, full-term neonates immediately after Caesarean section or normal delivery. BM and G-CSF mobilized PB cells were collected from normal healthy donors. All samples were collected after written informed consent, using protocols approved by the Investigational Review Boards at Osaka University and Oklahoma Medical Research Foundation. Mononuclear cells were separated by Ficoll-Paque PLUS (GE Healthcare Bio-Science AB, Uppsala, Sweden) or Lymphocyte Separation Medium (Mediatech, Inc., Manassas, VA) and centrifugation. Purification of CB and BM CD34^+^ cells was performed using Direct human CD34 Progenitor Cell Isolation Kit (Miltenyi Biotec, Auburn, CA). Human specimens were separately analyzed with respect to progenitor phenotypes to reveal individual to individual variability. Rare progenitor cells were mixed to have sufficient numbers to initiate culture experiments. Human mesenchymal stem cells were purchased from Lonza (Walkersville, MD), and maintained in Mesenchymal Stem Cell Growth Medium (MSCGM, Lonza). Flow cytometric analysis confirmed that the cultured hMSC expressed CD105, CD166, CD29, and CD44, but not CD14, CD34, or CD45.

### Co-cultures for human B lymphocytes

Co-cultures of CD34^+^ cells on hMSC were performed as previously described [30]. hMSC were seeded in 12-well tissue plates 1 or 2 days before setting up the co-cultures. Isolated CD34^+^ cells (2×10^3^ cells/well) were plated on sub-confluent hMSC layers in MSCGM in the presence of 10 ng/ml SCF and 5 ng/ml FL. Recombinant human SCF and FL proteins were purchased from R&D Systems, Inc. (Minneapolis, MN). Half of the culture medium was replaced with fresh medium containing the same cytokines twice a week.

### Stromal cell-free cultures for human B lymphocytes

Stromal cell-free cultures were performed as previously described [32]. The cultures were usually maintained in QBSF®60 (Quality Biological, Inc., Gaithersburg, MD) containing10% FCS, 100 U/ml penicillin, and 100 mg/ml streptomycin with indicated cytokines. Recombinant human IL-7 and G-CSF proteins were purchased from R&D Systems. Half of the culture medium was replaced with fresh medium containing the same cytokines once a week. The cytokines were used at the following concentrations: SCF, 10 ng/ml; FL, 5 ng/ml; G-CSF, 10 ng/ml; and IL-7, 5 ng/ml.

### Flow cytometry and cell sorting

Flow cytometric analysis was performed with a FACS Calibur or FACS LSRII (BD Biosciences Immunocytometry Systems, San Jose, CA) using standard multicolor immunofluorescent staining protocols. Mouse monoclonal Abs against the following human cell surface molecules were purchased: phycoerythrin (PE)-CD7, FITC-CD9, PE-CD10, allophycocyanin (APC)-CD10, APC-CD11c, FITC-CD14, PE-CD19, FITC-CD20, FITC-CD33, PE-CD34, APC-CD34, APC-CD90, PE-CD117, PE-CD123, PE-CD135, FITC-IgM and FITC-Ki67 from BD Bioscieinces/BD Pharmingen (Franklin Lakes, NJ); APC-CD22 from Bio Legend (San Diego, CA); phycoerythrin 5-succinimidylester (PC5)-CD19, PC5-CD24 from Beckman Coulter (Marseilles, France). In some experiments, CD34^+^ CD10^−^, CD34^+^ CD10^Lo^ and CD34^+^ CD10^Hi^ cells were sorted using a FACS Aria (BD Biosciences Immunocytometry Systems) and subjected to the cultures for human B lymphocytes. In this case, enriched CD34^+^ cells were stained with APC-CD34 and PE-CD10.

### Real-time quantitative PCR analysis of IgH gene rearrangement

A Genomic DNA was isolated from 1×10^4^ sorted subsets with a DNeasy Tissue Kit (QIAGEN, Valencia, CA). Taqman-based quantitative PCR was used to detect IgH gene rearrangement, using TaqMan Universal PCR Master Mix (Applied Biosystems, Foster City, CA). The probe was designed to detect one of the main D_H_ gene segment families (D_H_3–10) and J_H_4 gene segment, where D_H_-J_H_ rearrangement most frequently occurrs in humans (Applied Biosystems) [28]. Expression levels were determined by the internal control gene (C/Ebpα). Each sample was measured in triplicate, and the comparative threshold cycle method was used for relative quantification of gene expression. The primer sequences are available from the authors on request.

### RNA isolation and Real-time quantitative PCR analysis of gene expression

The mRNAs were isolated from sorted cells by using RNeasy Mini Kit (QIAGEN). The cDNAs were then prepared from DNase I-treated mRNA by using oligo(dT) and Moloney murine leukemia virus reverse transcriptase (Invitrogen, Carlsbad, CA). Reactions were quantified with fluorescent TaqMan technology. TaqMan primers and probes specific for indicated or S18 gene were used in the ABI750 sequence detection system (Applied Biosystems). Reactions were run at an annealing temperature of 60°C with 40 cycles. Each sample was measured in triplicate, and the comparative threshold cycle method was used for relative quantification of gene expression.

### BrdU assay

CD34^+^ CD10^−^, CD34^+^ CD10^Lo^ and CD34^+^ CD10^Hi^ cells were sorted and cultured in the presence of 10 ng/ml SCF, 5 ng/ml FL, and 10 ng/ml G-CSF for 4 days. They were pulsed with 10 mM BrdU for the final 48 hours. FITC BrdU Flow Kit purchased from BD Bioscieinces/BD Pharmingen was used.

### Statistical analyses

Student's t-test was performed to assess statistical differences. All results are shown as mean values ± SD.

## Supporting Information

Figure S1 The efficiency of B cell generation in culture depends on the source of progenitor cells. CD34^+^ cells were sorted from CB, G-CSF mobilized peripheral blood (G-PB) or BM, and cultured with hMSC in the presence of SCF and FL for 4 weeks. A representative analysis is shown (A). Numbers of total and CD10^+^ CD19^+^ B cells generated were calculated (B). Similar results were obtained in three independent experiments. Statistical significances were determined by unpaired two-tailed t test analysis: *, p<0.05 and **, p<0.01.
(0.45 MB TIF)

Figure S2 Comparison of B lymphopoiesis in mice and humans. Our new results and ones from the literature were used to construct a possible sequence of events in human B lymphocyte formation. Progressive down-regulation of c-Kit occurs from stem/early progenitor stages in mice, and our observations suggest that is also the case for humans. Three categories of human CD10^−^, CD10^Lo^ and CD10^Hi^ cells evaluated in the present study are positioned with respect to stem cells and lymphoid committed progenitors.
(0.25 MB TIF)
