# Supplementary figures and images for: The Density of CD10 Corresponds to Commitment and Progression in the Human B Lymphoid Lineage
Source: PLoS One. 2010 Sep 23;5(9):e12954. doi: 10.1371/journal.pone.0012954 (PMC2944886; doi:10.1371/journal.pone.0012954)

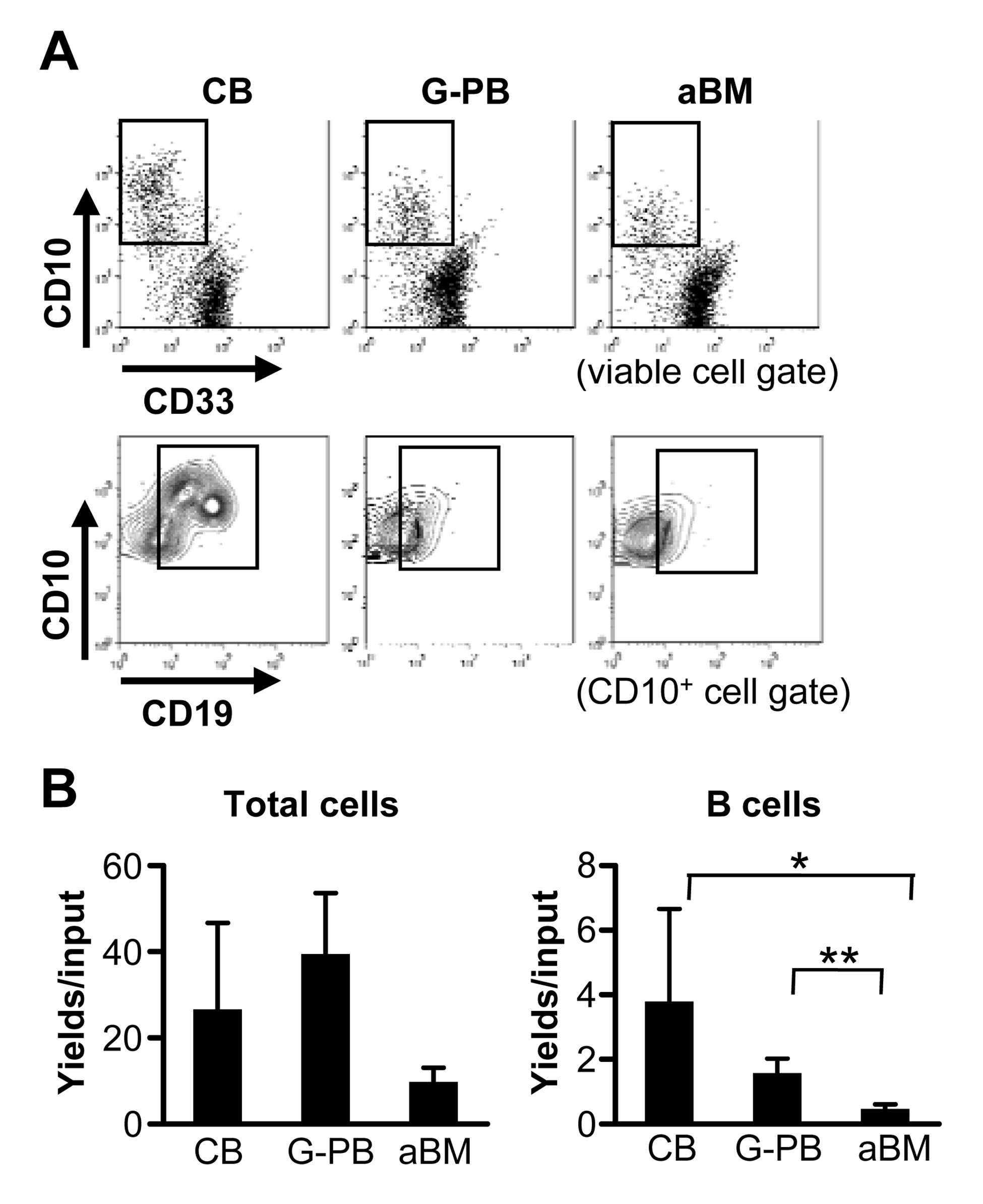

Supplement: Figure S1 — The efficiency of B cell generation in culture depends on the source of progenitor cells. CD34+ cells were sorted from CB, G-CSF mobilized peripheral blood (G-PB) or BM, and cultured with hMSC in the presence of SCF and FL for 4 weeks. A representative analysis is shown (A). Numbers of total and CD10+ CD19+ B cells generated were calculated (B). Similar results were obtained in three independent experiments. Statistical significances were determined by unpaired two-tailed t test analysis: *, p<0.05 and **, p<0.01. (0.45 MB TIF) [file pone.0012954.s001.tif]

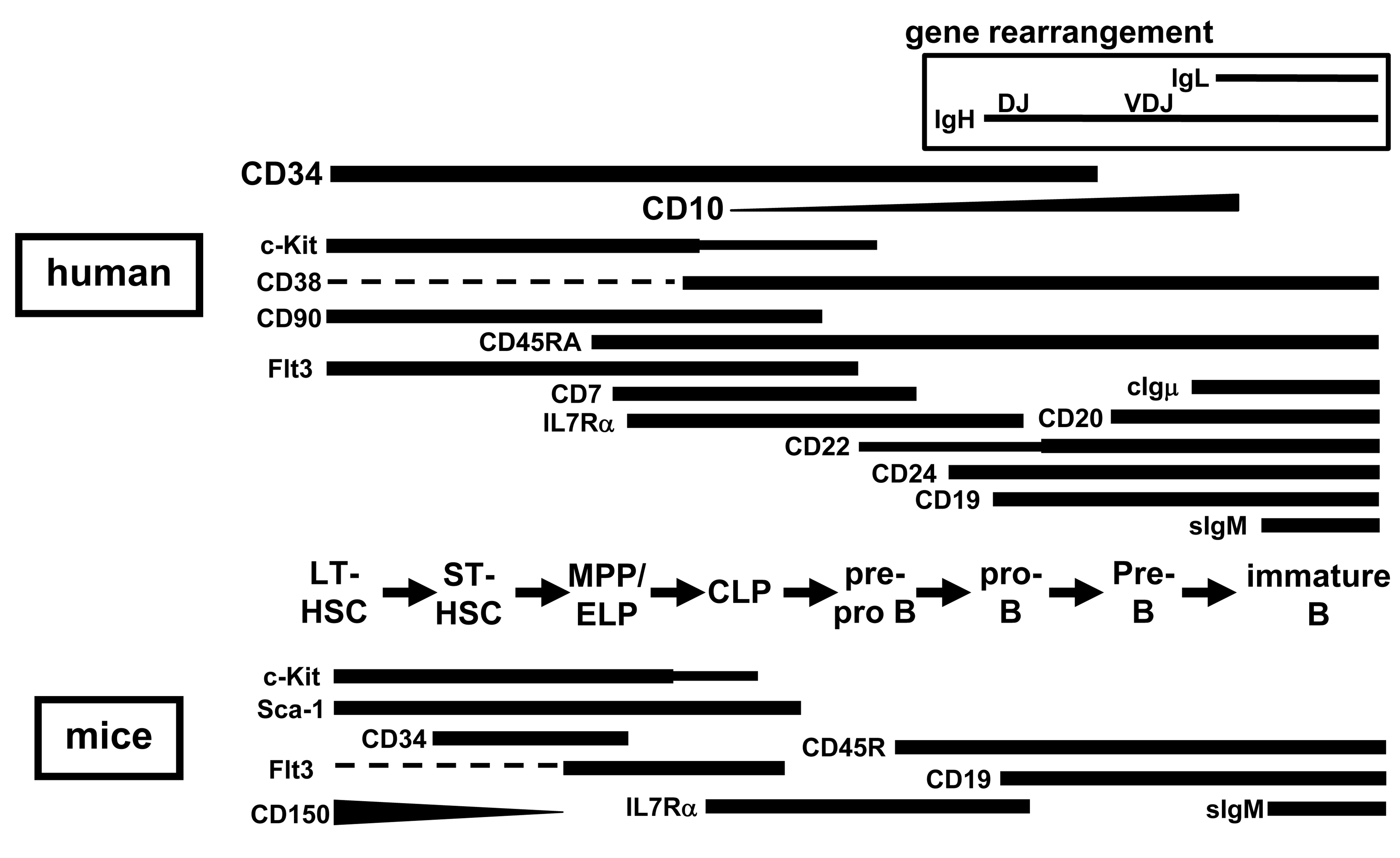

Supplement: Figure S2 — Comparison of B lymphopoiesis in mice and humans. Our new results and ones from the literature were used to construct a possible sequence of events in human B lymphocyte formation. Progressive down-regulation of c-Kit occurs from stem/early progenitor stages in mice, and our observations suggest that is also the case for humans. Three categories of human CD10−, CD10Lo and CD10Hi cells evaluated in the present study are positioned with respect to stem cells and lymphoid committed progenitors. (0.25 MB TIF) [file pone.0012954.s002.tif]
